# Does proton pump inhibitors use increase risk of digestive tumors?: A 2-sample Mendelian randomization study

**DOI:** 10.1097/MD.0000000000036085

**Published:** 2023-11-10

**Authors:** Ruiqi Zhao, Sen Lin, Mengyao Han, Zhimei Lin, Mengjiao Yu, Lisheng Peng

**Affiliations:** a The Fourth Clinical Medical College, Guangzhou University of Chinese Medicine, Shenzhen, China; b The Second Clinical Medical College, Guangzhou University of Chinese Medicine, Guangzhou, China; c Shenzhen Traditional Chinese Medicine Hospital, Shenzhen, Guangdong, China.

**Keywords:** digestive system tumors, mendelian randomization (MR), omeprazole, proton pump inhibitors (PPIs)

## Abstract

The objective of this study was to explore the causal relationship between the use of proton pump inhibitors (PPIs) and 16 types of digestive system tumors. We utilized a 2-sample Mendelian randomization (MR) approach to investigate this relationship. We obtained exposure and outcome data from the UK Biobank and the Finland Biobank, respectively. The genetic data used in the analysis were derived from genome-wide association studies (GWAS) studies conducted on European populations. We screened single nucleotide polymorphisms significantly associated with the use of omeprazole, a commonly used PPIs, as instrumental variables. We then performed MR analyses using the inverse variance weighting (IVW) method, MR-Egger regression, and the weighted median method to evaluate the causal effect of omeprazole use on the 16 types of digestive system tumors. Our MR analysis revealed a significant causal relationship between the use of omeprazole and pancreatic malignancies, but not with any other types of digestive system tumors. The IVW analysis showed an odds ratio of 4.33E-05 (95%CI: [4.87E-09, 0.38], *P* = .03) and the MR-Egger analysis showed an odds ratio of 5.81E-11 (95%CI: [2.82E-20, 0.12], *P* = .04). We found no significant heterogeneity or pleiotropy, and sensitivity analysis confirmed the robustness of our results. Furthermore, statistical power calculations suggested that our findings were reliable. Conclusion The use of PPIs is a protective factor for pancreatic malignancies, but no causal relationship has been found with other digestive system tumors.

## 1. Introduction

Proton pump inhibitors (PPIs) are a commonly used treatment for acute and chronic acid-related diseases of the digestive system, including gastroesophageal reflux disease, Zollinger-Ellison syndrome, peptic ulcer, upper gastrointestinal bleeding, and Helicobacter pylori infection. They work quickly and effectively to suppress gastric acid secretion. However, the potential adverse consequences of frequent and long-term PPIs use should be taken seriously, as they are widely used in clinical practice. The question of whether PPIs use increases the risk of developing digestive system tumors is a topic of debate within the international community.

A population-based study showed that long-term PPIs users had a 2.4-fold increased risk of gastric cancer compared with non-users. This increased risk was not observed among histamine 2-receptor antagonist users.^[1]^ In contrast, a Korean population-based cohort study found no association between PPIs use and an increased risk of gastric cancer.^[2]^ A meta-analysis suggested that the use of PPIs may be associated with an increased risk of pancreatic malignancies.^[3]^ On the other hand, another meta-analysis found no conclusive evidence of a link between PPIs use and pancreatic malignancies.^[4]^ A significant nested case-control study conducted in the United States found no association between exposure to proton pump inhibitors for more than 2 years and an increased risk of gastrointestinal tumors.^[5]^ In contrast, results from another comprehensive analysis suggested an association between PPIs use and a higher risk of gastric, pancreatic, and liver cancer, but not esophageal or colorectal cancer.^[6]^

Overall, the multiple observational studies and meta-analyses mentioned above have failed to reach a clear consensus, possibly due to differences in regions, populations, baseline characteristics, and follow-up times of various cohorts, which may confound the results and conclusions of these studies. Furthermore, most studies only demonstrate a correlation and do not establish a significant causal relationship. Therefore, the current evidence is insufficient to support concerns about the carcinogenic side effects of PPIs.

Mendelian randomization (MR) is a technique that utilizes genetic variations as instrumental variables to mitigate potential biases that may arise in observational clinical studies and account for confounding factors. This method is frequently employed to infer causal relationships between exposure and outcomes.^[7]^ This study employs 2-sample Mendelian randomization to examine the potential causal relationship between PPIs use and digestive system tumors, with the goal of providing a reference for future clinical medication.

## 2. Materials and methods

### 2.1. Study design

In the 23rd edition of the World Health Organization Model List of Essential Medicines, published in 2023, Omeprazole is distinguished as the sole PPIs included.^[8]^ Data from 2020 substantiates its prevalent use, with prescription counts surpassing 56 million. This volume situates Omeprazole as the eighth most frequently prescribed pharmaceutical in the United States.^[9,10]^ For this study, omeprazole was selected as the exposure factor, with benign and malignant tumors of the esophagus, stomach, small intestine, colon, rectum, anus and anal canal, liver and bile duct, and pancreas serving as the outcomes. Two-sample Mendelian randomization was utilized to analyze all data, using publicly available summary statistics from genome-wide association studies (GWAS).

### 2.2. The basic assumptions

**Figure 1.**
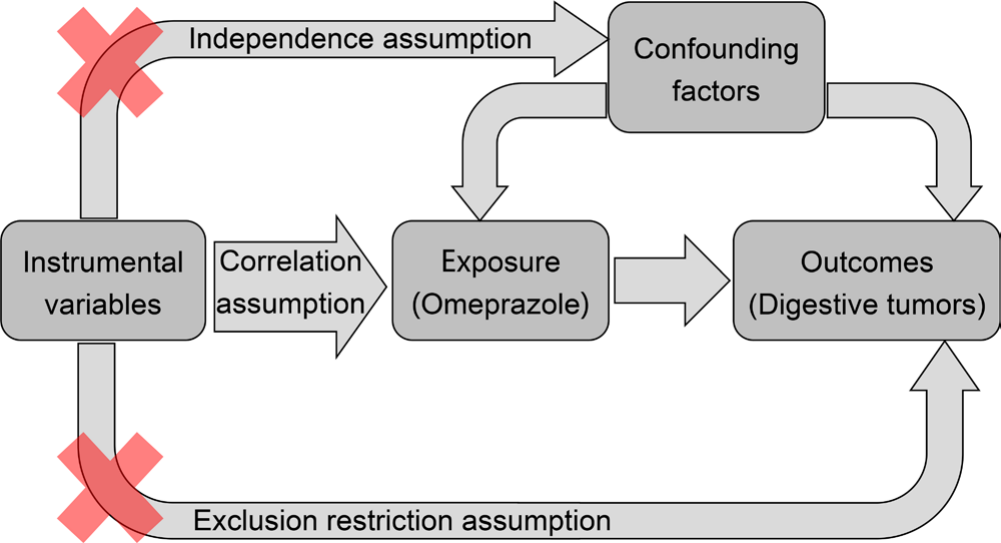
The schematic diagram of 2 sample mendelian randomization analysis.

To satisfy the 3 fundamental assumptions of 2-sample Mendelian randomization (as illustrated in Fig. 1), the following must be established: Correlation assumption, which requires the instrumental variable to be significantly associated with the exposure. Exclusion restriction assumption, which mandates that the instrumental variable is not associated with the outcome. Independence assumption, which necessitates that the instrumental variable is not associated with confounding factors.

The correlation assumption is evaluated using the formula *F* = BETA^2^exposure/SE^2^exposure,^[11]^ which yields an *F* value for each instrumental variable. An *F* value >10 indicates a strong instrumental variable, while a value <10 indicates a weak instrumental variable. To check the exclusion restriction and independence assumptions, each instrumental variable is examined in the PhenoScanner database^[12,13]^ for any associations with the outcome or confounding factors.

### 2.3. Data source

To mitigate potential biases based on ethnicity and gender, exposure and outcome data were obtained from GWAS data of European male and female populations. To reduce the risk of sample overlap, omeprazole data were obtained from the UK Biobank (http://www.nealelab.is/uk-biobank) with the data field “20003_1140865634,” while data for 16 types of digestive system tumors were obtained from the FinnGen Biobank in the IEU open GWAS database (https://gwas.mrcieu.ac.uk/datasets/?gwas_id__icontains=finn-b). To avoid potential confounding effects from other cancers, all digestive system tumors were excluded from other cancer types. Refer to Table 1 for further details.

**Table 1:** Summary of the GWAS included in this mendelian randomization study.

Phenotype	Nsamples	Ncases	Ncontrols	Number of SNPs
Benign neoplasm: Oesophagus	180,808	144	180,664	16,380,337
Benign neoplasm: Stomach	181,173	1252	179,921	16,380,339
Benign neoplasm: Small intestine	180,959	549	180,410	16,380,339
Benign neoplasm: Colon	183,354	7435	175,919	16,380,342
Benign neoplasm: Rectum	181,538	2108	179,430	16,380,339
Benign neoplasm: Anus and anal Canal	180,895	370	180,525	16,380,337
Benign neoplasm: Liver/Bile	180,839	264	180,575	16,380,337
Benign neoplasm: Pancreas	180,829	163	180,666	16,380,337
Malignant neoplasm: Oesophagus	174,238	232	174,006	16,380,304
Malignant neoplasm: Stomach	174,639	633	174,006	16,380,305
Malignant neoplasm: Small intestine	174,258	252	174,006	16,380,304
Malignant neoplasm: Colon	175,809	1803	174,006	16,380,317
Malignant neoplasm: Rectum	175,084	1078	174,006	16,380,307
Malignant neoplasm: Anus and anal Canal	174,114	108	174,006	16,380,303
Malignant neoplasm: Liver/Bile	174,310	304	174,006	16,380,303
Malignant neoplasm: Pancreas	174,611	605	174,006	16,380,306
Omeprazole	375,554	37,432	338,122	13,791,467

GWAS = genome-wide association studies, Nsamples = Number of samples; Ncase = Number of cases; Ncontrols = Number of controls; SNPs = single nucleotide polymorphisms.

### 2.4. Selection of instrumental variables

In this study, single nucleotide polymorphism (SNPs) were selected as instrumental variables based on their association with the exposure. Due to the constraint of the number of SNPs, those with *P* < 1.0E-5, a distance of 10,000 kb, linkage disequilibrium r^2^ < 0.001, and *F* > 10 were preferred, while missing and palindromic SNPs were excluded. Moreover, SNPs that were associated with the outcome were included in the statistical analysis. A total of 26 SNPs were used in the Mendelian randomization analysis, and detailed information for each SNP can be found in Table 2.

**Table 2:** The information for final single nucleotide polymorphisms.

SNP	EA	NEA	BETA	SE	*P*	MAF	*F*
rs74582510	G	A	−0.00802114	0.00178537	7.03E-06	0.0267214	20.18439
rs61784835	T	C	−0.00266877	0.000576468	3.67E-06	0.354462	21.43245
rs17391694	T	C	0.00356659	0.000791529	6.61E-06	0.140207	20.30358
rs77386202	T	C	0.0110266	0.00246712	7.85E-06	0.013506	19.97573
rs80154548	A	G	−0.00577222	0.001234	2.90E-06	0.0537769	21.88041
rs12477314	T	C	0.00310694	0.000690612	6.84E-06	0.197214	20.23939
rs3749313	G	A	0.00269177	0.000593113	5.67E-06	0.323762	20.59686
rs72680189	T	C	−0.00746049	0.00168187	9.18E-06	0.0273937	19.67659
rs6842546	C	T	−0.00304929	0.000677354	6.74E-06	0.221487	20.26591
rs115495176	A	G	0.00831285	0.00185751	7.63E-06	0.0222642	20.028
rs331082	T	C	0.00389715	0.000868578	7.23E-06	0.114342	20.13152
rs141915186	A	G	0.0123769	0.00261641	2.24E-06	0.0114862	22.37753
rs9266638	T	C	0.00302788	0.000608856	6.59E-07	0.286583	24.73137
rs9357772	A	C	0.00432472	0.000911972	2.12E-06	0.101874	22.48811
rs9479832	A	G	0.00315881	0.000698914	6.20E-06	0.209839	20.42676
rs73107935	A	G	0.00382152	0.000794695	1.52E-06	0.139793	23.12444
rs4240631	A	G	−0.00284302	0.000618405	4.28E-06	0.271092	21.13556
rs76982774	A	G	−0.0117291	0.00226833	2.33E-07	0.014882	26.73726
rs142022872	A	G	0.0118158	0.00262569	6.79E-06	0.0111466	20.25067
rs17201270	G	A	0.0077694	0.00165134	2.54E-06	0.028829	22.13614
rs17141685	G	A	0.00692139	0.00153124	6.18E-06	0.0333476	20.43149
rs2880898	G	A	0.00373759	0.000735951	3.80E-07	0.178276	25.79203
rs76479058	A	G	0.00909966	0.00202802	7.23E-06	0.018871	20.13288
rs7336891	C	A	−0.00276955	0.000553114	5.53E-07	0.472217	25.07201
rs494642	T	C	−0.00453664	0.00101416	7.70E-06	0.0817269	20.0104
rs384262	A	G	0.00283068	0.000560635	4.44E-07	0.413388.	25.49301

*P*, the significance level of SNP; The *F* statistic for each SNP was calculated as follows: *F* = BETA^2^exposure/SE^2^exposure.

BETA = beta exposure, EA = effect allele, MAF = minor allele frequency, NEA = non-effect allele, SE = standard error, SNP = single nucleotide polymorphism.

### 2.5. Statistical methods

To analyze the data in this study, 3 Mendelian randomization methods were utilized: the weighted median estimator, inverse variance weighting (IVW), and MR-Egger regression. The IVW method was used as the primary analysis method, while weighted median estimator and MR-Egger were employed as secondary methods. All analyses were conducted using the TwoSampleMR 0.5.6 package^[14,15]^ in R software (Version 4.2.1), with a significance level of α = 0.05.

### 2.6. Heterogeneity test, multiplicity analysis, and sensitivity analysis

To assess heterogeneity, the Cochran Q statistic was calculated using the IVW and MR-Egger methods, and the results were depicted on a funnel plot. *P* > .05 indicates the absence of heterogeneity. For multiplicity analysis, the intercept of the MR-Egger regression was evaluated using the mr_pleiotropy_test function. An intercept that tends to 0 and *P* > .05 suggest the absence of horizontal pleiotropy. Sensitivity analysis was conducted using the leave-one-out method, which involved excluding 1 SNP at a time and repeating the IVW analysis.

### 2.7. Statistical power

The statistical power of the MR analysis was assessed using the online tool mRND^[16]^ to validate the reliability of the results.

## 3. Result

### 3.1. Mendelian randomization analysis

**Figure 2.**
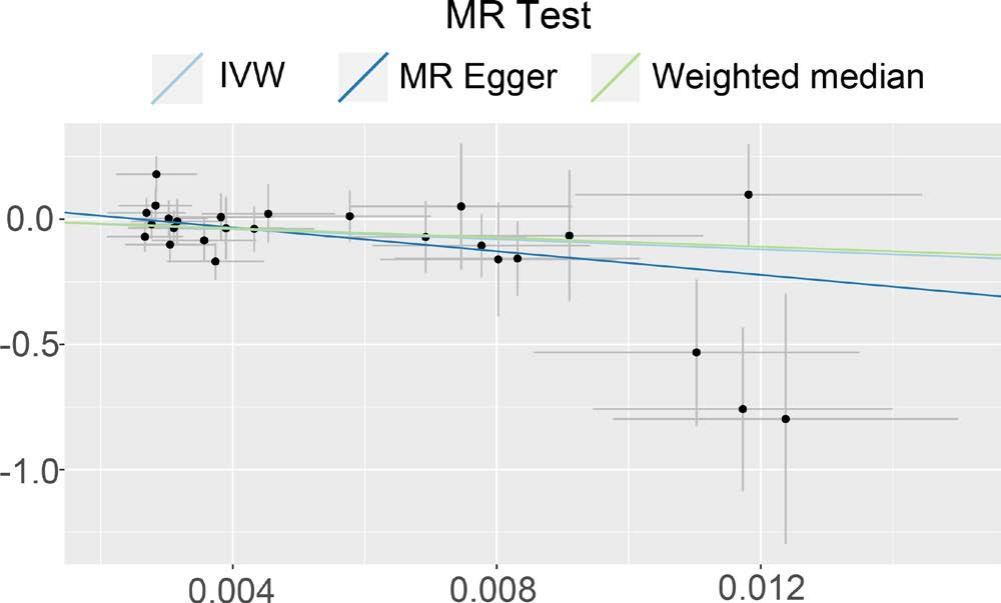
The scatter plot of 2 sample mendelian randomization analysis. MR = mendelian randomization; IVW = inverse variance weighting.

In this study, Mendelian randomization analysis was conducted separately for omeprazole and 16 benign and malignant digestive system tumors, including esophageal, gastric, small intestinal, colonic, rectal, anal and anal canal, pancreatic, hepatic, and biliary tract tumors. The results (Table 3) indicated that omeprazole had a causal relationship with only pancreatic malignancies among the 16 digestive system tumors, with an IVW (OR = 4.33E-05, 95%CI: [4.87E-09, 0.38], *P* = .03) and a MR Egger (OR = 5.81E-11, 95%CI: [2.82E-20, 0.12], *P* = .04). In other words, the use of omeprazole might be a protective factor for pancreatic malignancies, whereas there was no significant causal relationship with the other 15 digestive system tumors. Additionally, a scatter plot (Fig. 2) was generated using R software (Version 4.2.1) to visualize the potential causal relationship between omeprazole and pancreatic malignancies.

**Table 3:** Mendelian randomization analysis results

Outcome	Statistical methods	BETA	SE	*P*	OR
Benign neoplasm: Oesophagus	MR Egger	10.50	25.49	.68	3.64E + 04
	IVW	−10.67	10.60	.31	2.32E-05
	WME	−6.86	13.35	.61	1.05E-03
Benign neoplasm: Stomach	MR Egger	0.37	7.47	.96	1.44E + 00
	IVW	4.60	3.12	.14	9.91E + 01
	WME	2.54	4.75	.59	1.27E + 01
Benign neoplasm: Small intestine	MR Egger	−16.83	11.33	.15	4.92E-08
	IVW	−6.22	4.71	.19	1.99E-03
	WME	−4.95	6.56	.45	7.05E-03
Benign neoplasm: Colon	MR Egger	−5.87	3.26	.08	2.83E-03
	IVW	−0.01	1.37	>.99	9.92E-01
	WME	−1.46	1.87	.44	2.33E-01
Benign neoplasm: Rectum	MR Egger	−8.61	5.84	.15	1.82E-04
	IVW	−3.15	2.44	.20	4.29E-02
	WME	−3.09	3.40	.36	4.56E-02
Benign neoplasm: Anus and anal Canal	MR Egger	11.61	13.71	.41	1.10E + 05
	IVW	−0.71	5.72	.90	4.94E-01
	WME	3.47	7.68	.65	3.23E + 01
Benign neoplasm: Liver/Bile	MR Egger	0.47	19.39	.98	1.61E + 00
	IVW	3.35	7.92	.67	2.86E + 01
	WME	−3.40	9.56	.72	3.34E-02
Benign neoplasm: Pancreas	MR Egger	−18.09	20.62	.39	1.39E-08
	IVW	−4.89	8.59	.57	7.52E-03
	WME	−16.74	12.02	.16	5.38E-08
Malignant neoplasm: Oesophagus	MR Egger	−18.57	17.30	.29	8.61E-09
	IVW	−4.12	7.22	.57	1.63E-02
	WME	−11.25	9.87	.25	1.30E-05
Malignant neoplasm: Stomach	MR Egger	−9.79	10.59	.36	5.62E-05
	IVW	3.56	4.40	.42	3.51E + 01
	WME	−0.64	6.08	.92	5.27E-01
Malignant neoplasm: Small intestine	MR Egger	1.98	16.62	.91	7.25E + 00
	IVW	−11.40	6.91	.10	1.12E-05
	WME	−7.16	9.48	.45	7.77E-04
Malignant neoplasm: Colon	MR Egger	4.02	7.76	.61	5.56E + 01
	IVW	1.16	3.18	.71	3.20E + 00
	WME	−0.39	3.91	.92	6.80E-01
Malignant neoplasm: Rectum	MR Egger	6.14	8.54	.48	4.64E + 02
	IVW	−4.34	3.61	.23	1.30E-02
	WME	−3.31	4.89	.50	3.66E-02
Malignant neoplasm: Anus and anal Canal	MR Egger	5.62	25.30	.83	2.75E + 02
	IVW	−11.94	10.53	.26	6.50E-06
	WME	−18.09	14.02	.20	1.39E-08
Malignant neoplasm: Liver/Bile	MR Egger	1.54	15.10	.92	4.66E + 00
	IVW	8.97	6.29	.15	7.86E + 03
	WME*	17.47	8.67	.04	3.87E + 07
Malignant neoplasm: Pancreas	MR Egger*	−23.57	10.94	.04	5.81E-11
	IVW*	−10.05	4.64	.03	4.33E-05
	WME	−9.18	6.25	.14	1.03E-04

BETA = beta exposure, IVW = inverse variance weighting, MR = mendelian randomization, OR = odds ratio; *p*, the significance level of mendelian randomization analysis results, SE = standard error, WME = weighted median estimator.

* *P <*.05.

### 3.2. Heterogeneity test, multiplicity analysis, and sensitivity analysis

**Figure 3.**
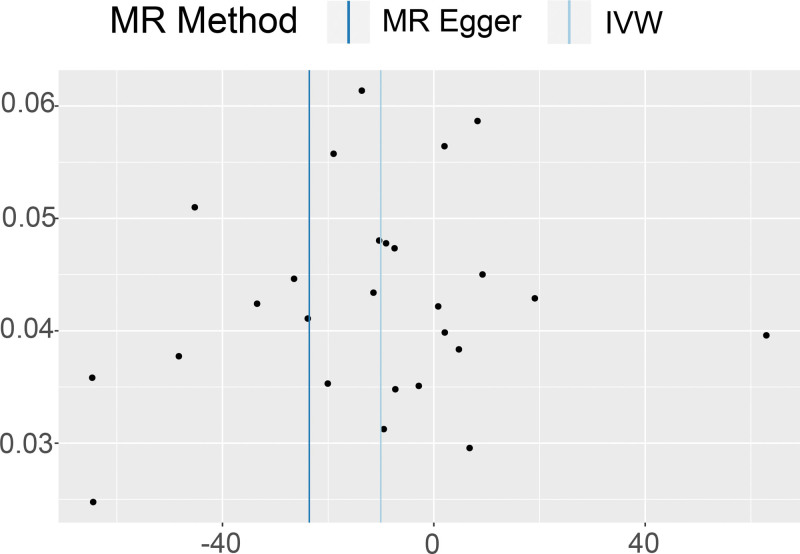
The funnel plot of 2 sample mendelian randomization analysis. MR = mendelian randomization; IVW = inverse variance weighting.

**Figure 4.**
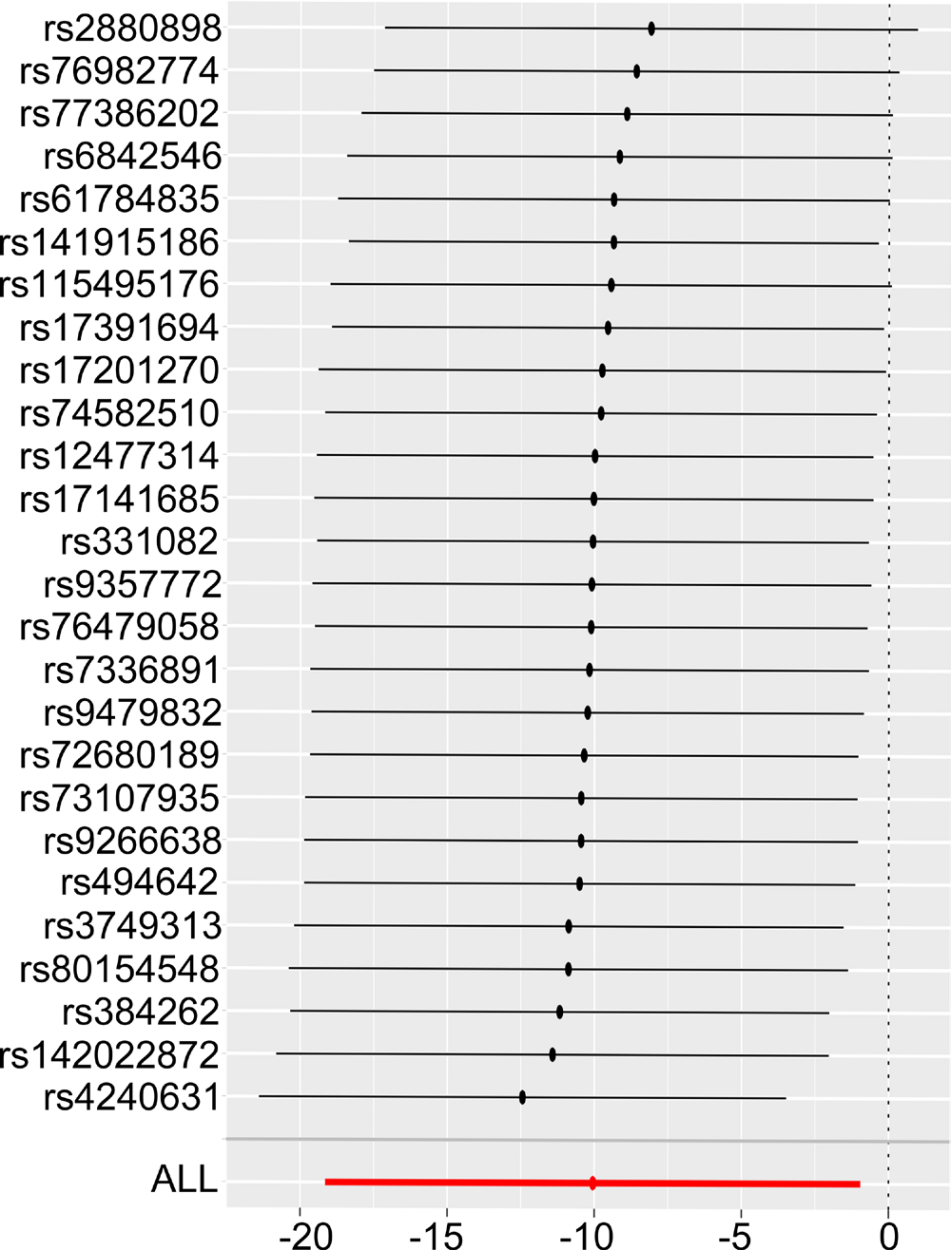
The forest map based on the analysis result of “leave- one- out method”.

Both the IVW method (Q = 26.59609, *P* = .38) and the MR-Egger method (Q = 24.69430, *P* = .42) did not reveal any significant heterogeneity in the heterogeneity test. A funnel plot (Fig. 3), drawn using R software (Version 4.2.1), showed that the points in the plot were evenly distributed, without indicating significant heterogeneity. In the scatter plot (Fig. 2), the Y-axis intercept of the MR-Egger regression line was 0.06021148, tending to 0, and *P* = .19, suggesting the absence of significant horizontal pleiotropy in this study. The results remained stable after excluding SNPs one by one using the leave-one-out method, and no significant impact on the results was observed after excluding any SNP (Fig. 4).

### 3.3. Statistical power

The relevant data were imported into the mRND online tool, and the statistical power was computed as Power = 1, indicating that the MR analysis results of omeprazole and pancreatic malignancies were highly reliable.

## 4. Discussion

This study utilized the Mendelian randomization method to investigate the causal relationship between omeprazole and the incidence of 16 digestive system tumors. The results indicated that omeprazole use was a protective factor against pancreatic malignancies, as supported by the IVW method (OR = 4.33E-05, 95%CI: [4.87E-09, 0.38], *P* = .03) and the MR Egger method (OR = 5.81E-11, 95%CI: [2.82E-20, 0.12], *P* = .04). The results were robust, as evidenced by the heterogeneity test, pleiotropy analysis, sensitivity analysis, and statistical power calculation. In summary, taking omeprazole can reduce the incidence of pancreatic malignancies, and there was no significant causal relationship with the remaining 15 digestive system tumors.

As mentioned previously, observational studies and meta-analyses both domestically and internationally have not yielded a definitive conclusion regarding the causal relationship between the use of PPIs and digestive system tumors. With respect to biological mechanisms, Andrej Udelnow et al found that omeprazole can regulate the autophagy of pancreatic malignancies cells, either directly or indirectly, through the lysosomal transport pathway, thereby suppressing the proliferation of pancreatic malignancies cells.^[17]^ According to Matthew A Huber et al, hypergastrinemia resulting from PPIs could be the cause of pancreatic intraepithelial neoplasia and pancreatic malignancies, and this hypothesis was confirmed through animal experiments.^[18]^ PPIs might increase the risk of digestive system tumor occurrence by elevating blood gastrin concentration, increasing nitrite concentration, and enhancing the toxicity of chemotherapy drugs. Conversely, PPIs could act as a protective factor for digestive system tumors by modifying the tumor microenvironment, regulating tumor cell pH value, promoting tumor cell apoptosis, inhibiting tumor cell growth, improving tumor cell drug resistance, and other functions. In summary, PPIs could be a double-edged sword for the development of digestive system tumors.

This study employed genetic variation as an instrumental variable to mitigate biases and reverse causality resulting from confounding factors such as acquired smoking, drinking, disease, and lifestyle habits from a genetic perspective. To effectively avoid racial bias and sample overlap, this article utilized public GWAS databases and selected individuals from the same race in different regions for both exposure and outcome. The large sample size of GWAS data provided robust support for statistical power. Therefore, the use of Mendelian randomization methods to examine the causal relationship between exposure and outcome could yield more reliable evidence than previous traditional observational studies.

However, this study is not without limitations. Firstly, the study population was restricted to European populations, and thus, the generalizability of the findings to other races is unclear. Secondly, Mendelian randomization assumes a linear relationship between exposure and outcome; if this assumption is violated, the results may not be applicable. Thirdly, due to database limitations, detailed information on omeprazole use, such as dosage, duration, and mode of administration (oral or intravenous injection), was unavailable for detailed subgroup analysis. Finally, the causal relationship between omeprazole and pancreatic malignancies identified in this study is only theoretical and does not elucidate its biological mechanism.

## 5. Conclusion

In summary, previous studies have often regarded PPIs use as a risk factor for digestive system tumors. This study employed 2-sample Mendelian randomization methods with genetic variation as an instrumental variable to investigate the causal relationship between omeprazole use and the incidence of 16 digestive system tumors. The study found evidence that omeprazole use could reduce the risk of pancreatic malignancies incidence, but no causal relationship was detected between omeprazole use and esophageal or stomach tumors, among other types of digestive system tumors. Therefore, the evidence for the long-term use of PPIs increasing the risk of developing digestive system tumors remains inconclusive, and additional research is needed to explore its potential mechanisms for preventing tumor occurrence. This study has significant clinical implications for the safe use of PPIs and can provide new insights for future clinical and cell-based research. Larger-scale prospective cohort studies, randomized controlled experiments, or cell experiments are necessary to generalize and apply these findings.

## Author contributions

**Data curation:** Ruiqi Zhao, Mengyao Han, Zhimei Lin.

**Funding acquisition:** Lisheng Peng.

**Investigation:** Zhimei Lin, Mengjiao Yu.

**Methodology:** Ruiqi Zhao, Sen Lin, Lisheng Peng.

**Project administration:** Lisheng Peng.

**Resources:** Ruiqi Zhao, Mengyao Han, Mengjiao Yu.

**Software:** Ruiqi Zhao, Mengyao Han.

**Supervision:** Sen Lin, Mengyao Han, Zhimei Lin, Mengjiao Yu, Lisheng Peng.

**Validation:** Sen Lin, Mengyao Han.

**Visualization:** Ruiqi Zhao.

**Writing – original draft:** Ruiqi Zhao.

**Writing – review & editing:** Ruiqi Zhao, Sen Lin, Lisheng Peng.

## References

[1] Cheung KS Chan EW Wong AYS. Long-term proton pump inhibitors and risk of gastric cancer development after treatment for *Helicobacter pylori*: a population-based study. Gut. 2018;67:28–35. 29089382 10.1136/gutjnl-2017-314605

[2] Gong EJ Bang CS Kim DK. Use of proton pump inhibitors and the risk for the development of gastric cancers: a nationwide population-based cohort study using balanced operational definitions. Cancers (Basel). 2022;14:5172. 36291956 10.3390/cancers14205172 PMC9600864

[3] Hong HE Kim AS Kim MR. Does the use of proton pump inhibitors increase the risk of pancreatic cancer? A systematic review and meta-analysis of epidemiologic studies. Cancers (Basel). 2020;12:2220. 32784492 10.3390/cancers12082220 PMC7463819

[4] Laoveeravat P Thavaraputta S Vutthikraivit W. Proton pump inhibitors and histamine-2 receptor antagonists on the risk of pancreatic cancer: a systematic review and meta-analysis. QJM. 2020;113:100–7. 31503318 10.1093/qjmed/hcz234

[5] Lee JK Merchant SA Schneider JL. Proton pump inhibitor use and risk of gastric, colorectal, liver, and pancreatic cancers in a community-based population. Am J Gastroenterol. 2020;115:706–15. 32205645 10.14309/ajg.0000000000000591

[6] Zeng R Cheng Y Luo D. Comprehensive analysis of proton pump inhibitors and risk of digestive tract cancers. Eur J Cancer. 2021;156:190–201. 34481369 10.1016/j.ejca.2021.07.030

[7] Davey Smith G Hemani G. Mendelian randomization: genetic anchors for causal inference in epidemiological studies. Hum Mol Genet. 2014;23(R1):R89–98. 25064373 10.1093/hmg/ddu328 PMC4170722

[8] World Health Organization Model List of Essential Medicines – 23rd List, 2023. In: The selection and use of essential medicines 2023: Executive summary of the report of the 24th WHO Expert Committee on the Selection and Use of Essential Medicines, 24 – 28 April 2023. Geneva: World Health Organization; 2023 (WHO/MHP/HPS/EML/2023.02). Licence: CC BYNC-SA 3.0 IGO. https://www.who.int/publications/i/item/WHO-MHP-HPS-EML-2023.02

[9] The Top 300 of 2020. https://clincalc.com/DrugStats/Top300Drugs.aspx

[10] Omeprazole - Drug Usage Statistics, ClinCalc DrugStats Database. https://clincalc.com/DrugStats/Drugs/Omeprazole

[11] Bowden J Del Greco M F Minelli C. Assessing the suitability of summary data for two-sample Mendelian randomization analyses using MR-Egger regression: the role of the I2 statistic. Int J Epidemiol. 2016;45:1961–74. 27616674 10.1093/ije/dyw220 PMC5446088

[12] Staley JR Blackshaw J Kamat MA. PhenoScanner: a database of human genotype-phenotype associations. Bioinformatics. 2016;32:3207–9. 27318201 10.1093/bioinformatics/btw373 PMC5048068

[13] Kamat MA Blackshaw JA Young R. PhenoScanner V2: an expanded tool for searching human genotype-phenotype associations. Bioinformatics. 2019;35:4851–3. 31233103 10.1093/bioinformatics/btz469 PMC6853652

[14] Hemani G Tilling K Davey Smith G. Orienting the causal relationship between imprecisely measured traits using GWAS summary data. PLoS Genet. 2017;13:e1007081. 29149188 10.1371/journal.pgen.1007081 PMC5711033

[15] Hemani G Zheng J Elsworth B. The MR-Base platform supports systematic causal inference across the human phenome. Elife. 2018;7:e34408. 29846171 10.7554/eLife.34408 PMC5976434

[16] Brion MJA Shakhbazov K Visscher PM. Calculating statistical power in Mendelian randomization studies. Int J Epidemiol. 2013;42:1497–501. 24159078 10.1093/ije/dyt179 PMC3807619

[17] Udelnow A Kreyes A Ellinger S. Omeprazole inhibits proliferation and modulates autophagy in pancreatic cancer cells. PLoS One. 2011;6:e20143. 21629657 10.1371/journal.pone.0020143 PMC3101238

[18] Huber MA Nadella S Cao H. Does chronic use of high dose proton pump inhibitors increase risk for pancreatic cancer? Pancreas. 2022;51:1118–27. 37078934 10.1097/MPA.0000000000002145 PMC10119745

